# African swine fever virus hijacks host pyrimidine metabolism to promote viral replication

**DOI:** 10.1128/jvi.00985-25

**Published:** 2025-10-31

**Authors:** Zebu Song, Yilin Chen, Hui Guo, Guihong Zhang, Lang Gong, Zezhong Zheng

**Affiliations:** 1 Guangdong Provincial Key Laboratory of Zoonosis Prevention and Control, College of Veterinary Medicine, South China Agricultural University 12526, Guangzhou, China; 2 African Swine Fever Regional Laboratory of China (Guangzhou), South China Agricultural University 12526, Guangzhou, China; 3 Key Laboratory of Animal Vaccine Development, Ministry of Agriculture and Rural Affairs, Guangzhou, China; 4 Wen’s Food Group, Yunfu, China; Northwestern University Feinberg School of Medicine, Chicago, Illinois, USA

**Keywords:** African swine fever virus, pyrimidine metabolism, glutamine, aspartate, metabolic hijacking

## Abstract

African swine fever (ASF) is a highly contagious disease of pigs caused by the African swine fever virus (ASFV), posing a significant threat to global swine production. As an obligate intracellular parasite, ASFV relies on host metabolic networks to fulfill its replication requirements. However, the precise mechanisms by which it manipulates nucleotide metabolism remain unclear. In this study, untargeted metabolomic analysis of ASFV-infected porcine alveolar macrophages revealed significant perturbations in purine and pyrimidine metabolism, glycolysis, the pentose phosphate pathway (PPP), and the glutamate and aspartate metabolic pathways. Functional validation demonstrated that ASFV depends on *de novo* pyrimidine biosynthesis for viral genome replication. Notably, ASFV employs a dual strategy to sustain the supply of nucleotide precursors: (i) it hijacks the PPP to generate ribose-5-phosphate and NADPH for redox balance, and (ii) it enhances glutamine uptake and catabolism to provide the nitrogen and carbon needed for nucleotide biosynthesis and tricarboxylic acid cycle replenishment. Furthermore, although aspartate is essential for pyrimidine synthesis, ASFV circumvents dependence on extracellular aspartate by activating a cytosolic GOT1-mediated synthesis pathway. Collectively, these findings elucidate how ASFV reprograms host nucleotide metabolism to support its replication, offering new insights into virus–host metabolic interactions and identifying potential targets for antiviral therapy.

**IMPORTANCE:**

African swine fever (ASF) is a devastating disease that causes substantial economic losses in the global pig industry. This study demonstrates that the African swine fever virus (ASFV) reprograms host cell metabolism to produce the essential building blocks required for its replication. Specifically, ASFV manipulates host nucleotide biosynthetic pathways to secure both the substrates for DNA synthesis and the reducing power necessary to mitigate oxidative stress. Elucidating these metabolic interactions not only deepens understanding of ASFV pathogenesis but also highlights promising metabolic targets for antiviral therapy. By elucidating how ASFV hijacks nucleotide biosynthesis within infected cells, our findings pave the way for innovative strategies to combat ASF.

## INTRODUCTION

African swine fever (ASF), caused by the African swine fever virus (ASFV), is a highly contagious disease that has caused devastating economic losses in the global swine industry (1). ASFV is a large, double-stranded DNA virus with a complex genome that relies heavily on the host metabolic network for replication. Recent advances in virology have highlighted a universal strategy known as “metabolic hijacking,” in which viruses reprogram host metabolic networks to redirect resources toward viral propagation (2, 3). Emerging evidence suggests that ASFV alters host energy metabolism and amino acid utilization to support its replication cycle (4). In this context, understanding how ASFV manipulates host metabolism is critical not only for elucidating its replication mechanisms but also for identifying new antiviral targets.

Nucleotide metabolism is a prime example of a critical host pathway exploited by viruses. Nucleotides, the basic building blocks of both DNA and RNA, are indispensable for viral genome replication. Several viruses have been shown to enhance their replication by activating either the *de novo* nucleotide biosynthesis or the salvage pathways (5). However, it remains unclear whether ASFV modulates host nucleotide metabolism to meet its replication requirements and which mechanisms or key regulatory nodes are involved.

Nucleotides consist of a nitrogenous base, a pentose sugar, and phosphate groups. Glucose is funneled into the pentose phosphate pathway (PPP), which produces ribose-5-phosphate (R5P), a key precursor for nucleotide biosynthesis. PPP comprises an oxidative branch that generates NADPH—crucial for maintaining cellular redox homeostasis—and a non-oxidative branch that interconverts sugars (6). Several viruses have been reported to hijack the oxidative PPP (oxPPP) to secure the necessary building blocks for replication (7–9). Moreover, NADPH generated by the oxPPP enhances viral antioxidant capacity, thereby facilitating viral replication (10). Importantly, our prior work revealed that intracellular reactive oxygen species (ROS) levels increase significantly following ASFV infection (11), suggesting a potential role for oxPPP-derived NADPH in counteracting oxidative stress during ASFV replication.

In addition to glucose metabolism, amino acid metabolism plays a pivotal role in nucleotide biosynthesis. Glutamine is a key amino acid and the second major energy substrate after glucose. As a vital source of both carbon and nitrogen, glutamine can directly donate its γ-nitrogen for nucleotide synthesis once internalized. Furthermore, it can be converted into glutamate and subsequently into α-ketoglutarate (α-KG) to fuel the tricarboxylic acid (TCA) cycle or donate nitrogen via transamination for the synthesis of other non-essential amino acids (12). Cellular glutamine uptake is mediated by solute carrier (SLC) transporters, including SLC1A5, SLC38A1, and SLC38A2 (13). Recent studies have shown that viral infection can activate these glutamine transporters, thereby accelerating glutamine uptake (14) and upregulating relevant metabolic enzymes to enhance glutamine catabolism for nucleotide biosynthesis (15). However, whether ASFV similarly enhances glutamine uptake and catabolism remains to be fully elucidated.

Aspartate, a non-essential amino acid, is an indispensable substrate for constructing the pyrimidine ring and thus plays a key role in nucleotide synthesis and cell proliferation (16). Aspartate can be acquired through extracellular uptake or *de novo* synthesis. *De novo* aspartate synthesis is primarily driven by TCA cycle replenishment: mitochondrial oxaloacetate (OAA) is produced and converted into aspartate by mitochondrial GOT2, or alternatively, cytosolic GOT1 converts OAA into aspartate through reductive glutamine metabolism. When mitochondrial-dependent aspartate biosynthesis is impaired, GOT1 plays a crucial role in maintaining aspartate production and supporting cell proliferation (17). Previous studies have reported that defects in endogenous aspartate synthesis can suppress tumor growth (18), and Liu et al. found that foot-and-mouth disease virus enhances aspartate uptake via the SLC38A8 transporter to promote its replication (19). However, the role of aspartate in ASFV replication remains unclear, and further investigation is needed to elucidate how ASFV utilizes aspartate to support its life cycle.

In this study, we investigate how ASFV reprograms host nucleotide metabolism, focusing on the PPP, glutamine uptake and catabolism, and aspartate biosynthesis from a metabolic perspective. Our findings provide new insights into the metabolic interplay between ASFV and host cells and identify potential targets within nucleotide metabolism.

## RESULTS

### ASFV infection remodels nucleotide metabolic networks to potentially facilitate replication

**Fig 1:**
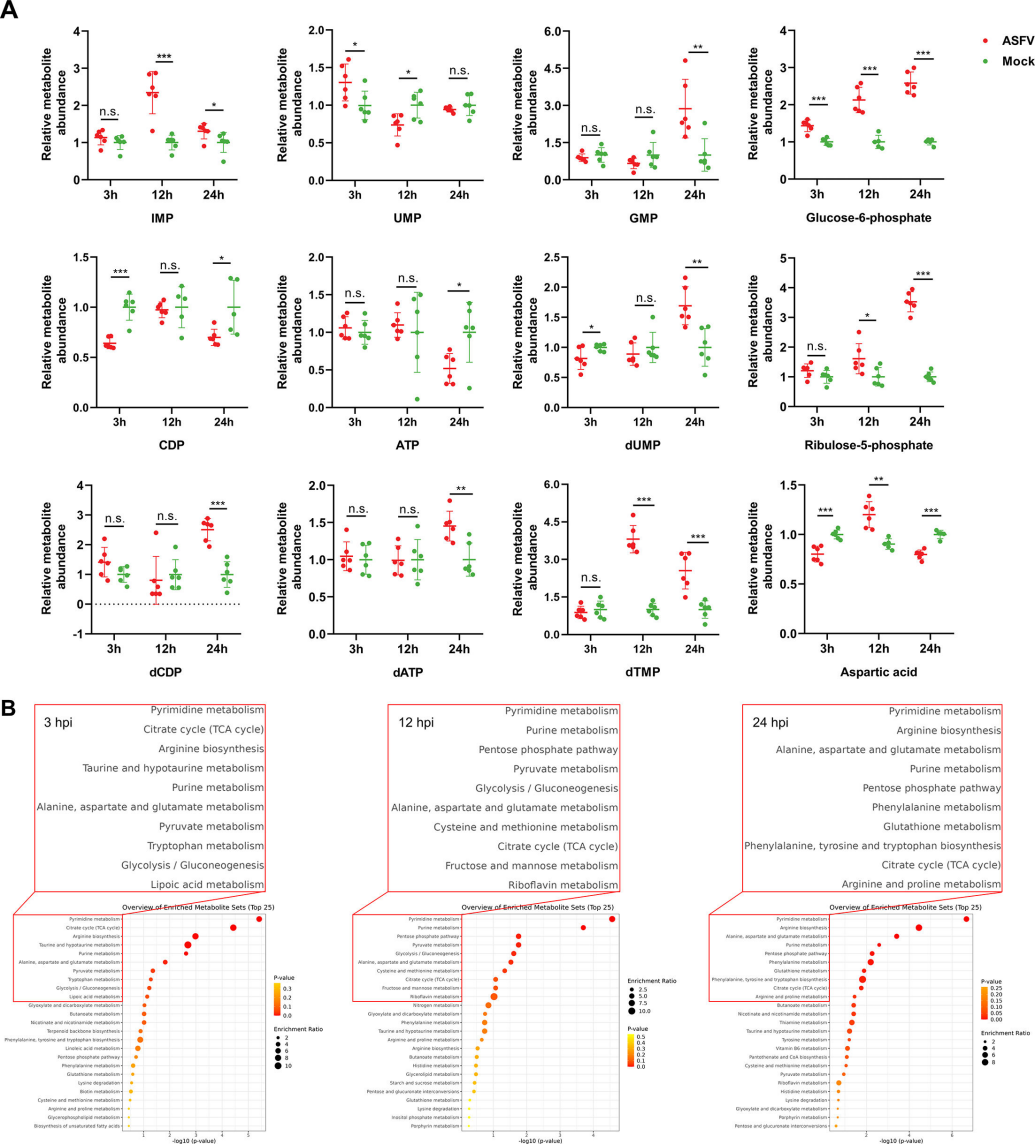
ASFV infection (multiplicity of infection [MOI] = 1) remodels host nucleotide metabolic networks. (**A**) ASFV infection induces metabolic changes in substances related to nucleotide metabolism (*n* = 6 independent biological replicates). (**B**) Pathway enrichment analysis of metabolite differences at various time points following ASFV infection. Statistical significance is indicated as follows: *P < 0.05, **P < 0.01, ***P < 0.001, n.s. = no significant difference (*P* ≥ 0.05).

Nucleotides play a critical role in viral replication, and the metabolic pathways governing their synthesis have become key targets in antiviral drug development (20). To investigate ASFV-induced metabolic perturbations, we reanalyzed our previous untargeted metabolomics data from ASFV-infected porcine alveolar macrophages (PAMs) across different infection stages (see the supplemental material). Notably, ASFV infection caused stage-specific accumulation and depletion of nucleotide precursors, including R5P and aspartate, as well as intermediates associated with nucleotide biosynthesis (Fig. 1A). These findings suggest that ASFV may facilitate its replication by modulating host nucleotide metabolic pathways. To further examine these changes, we performed pathway enrichment analysis using MetaboAnalyst version 5.0 (www.metaboanalyst.ca) to systematically identify pathways affected by ASFV infection. As shown in Fig. 1B, purine and pyrimidine metabolism pathways were consistently enriched across all infection stages (3, 12, and 24 hours post-infection [hpi]), along with related pathways such as the PPP and aspartate metabolism. Collectively, this spatiotemporal metabolic rewiring positions nucleotide metabolism as a central element of ASFV replication, highlighting potential targets for therapeutic intervention.

### ASFV replication selectively depends on pyrimidine nucleotide synthesis

**Fig 2:**
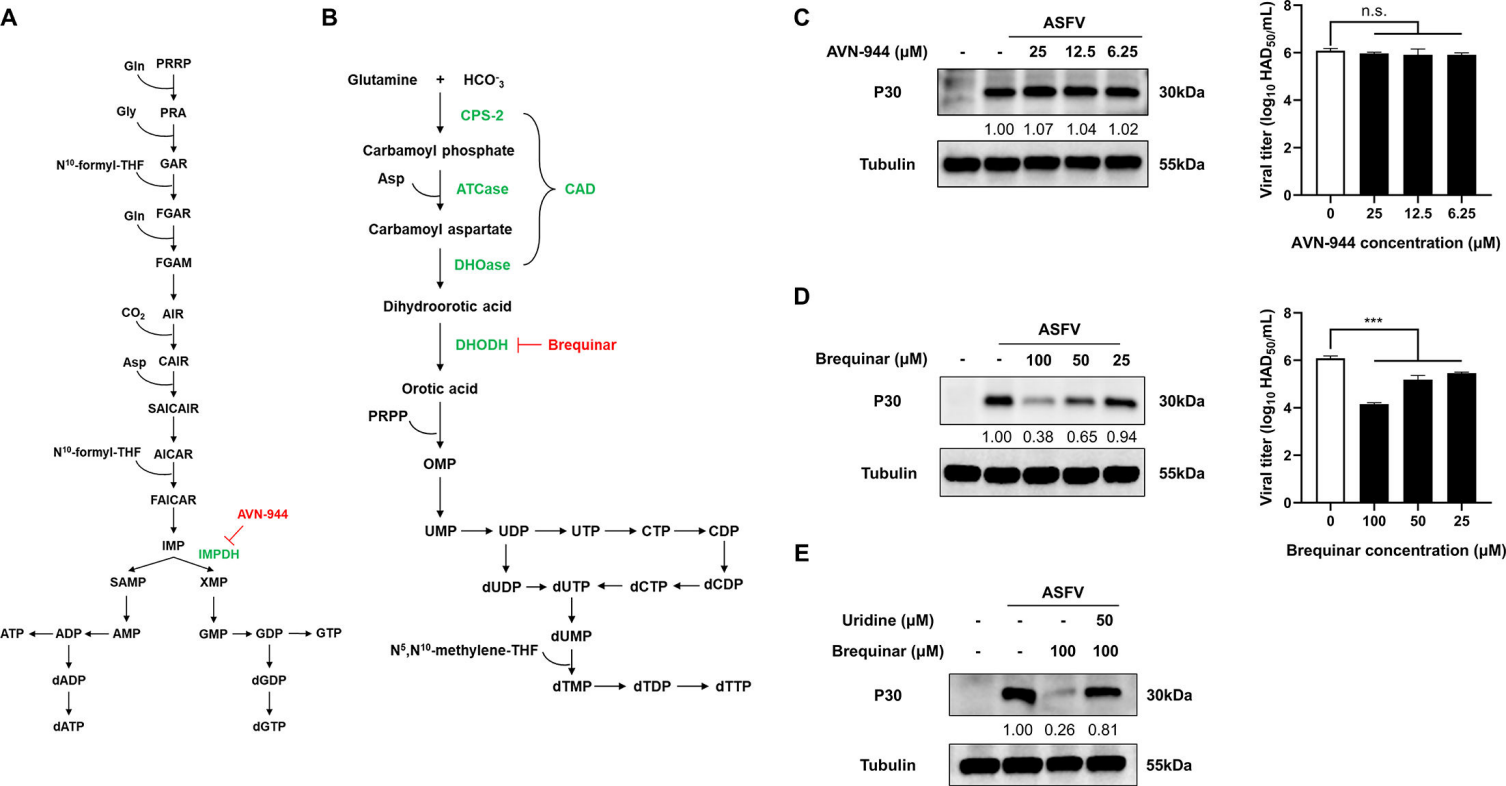
ASFV replication selectively depends on pyrimidine nucleotide synthesis. (**A**) Schematic diagram of the *de novo* synthesis pathway of purine nucleotides and the target sites of AVN-944, an inhibitor of IMPDH, a key enzyme in the purine pathway. (**B**) Schematic diagram of the *de novo* synthesis pathway of pyrimidine nucleotides and the target site of brequinar, an inhibitor of dihydroorotate dehydrogenase, a key enzyme in the pyrimidine pathway. (**C**) Western blot and virus titration analysis showing that treatment with AVN-944 does not alter ASFV replication in infected PAMs. (**D**) Western blot and virus titration analysis showing that treatment with brequinar inhibits ASFV replication in infected cells. (**E**) Exogenous uridine supplementation restores ASFV-P30 protein levels suppressed by brequinar. For all experiments, PAMs were infected with ASFV at MOI = 1, and cell lysates were collected at 24 hpi for Western blotting. Statistical significance is indicated as follows: *P < 0.05, **P < 0.01, ***P < 0.001, n.s. = no significant difference (*P* ≥ 0.05).

To determine whether ASFV replication relies on *de novo* synthesis of purine, pyrimidine, or both types of nucleotides, we evaluated the effects of AVN-944, an inhibitor of inosine monophosphate dehydrogenase (IMPDH, a key enzyme in the purine pathway), and brequinar, an inhibitor of dihydroorotate dehydrogenase (DHODH, a key enzyme in the pyrimidine pathway), on ASFV replication. As shown in Fig. 2C and Fig. S2A and B, various concentrations of AVN-944 did not affect the replication of the ASFV, whereas brequinar inhibited ASFV replication in a dose-dependent manner (Fig. 2D; Fig. S2C and D). To further determine whether brequinar suppresses ASFV replication by depleting intracellular pyrimidine levels, we supplemented infected cells with exogenous uridine to evaluate whether ASFV replication could be rescued via the pyrimidine salvage pathway. Notably, exogenous uridine restored ASFV replication reduced by brequinar treatment, suggesting that brequinar inhibited ASFV replication by depleting intracellular pyrimidine pools (Fig. 2E; Fig. S2E). These findings demonstrate that ASFV replication critically depends on pyrimidine nucleotide biosynthesis while being independent of *de novo* purine synthesis under the conditions tested.

### PPP coordinates antiviral redox balance and nucleotide metabolism to promote ASFV replication

**Fig 3:**
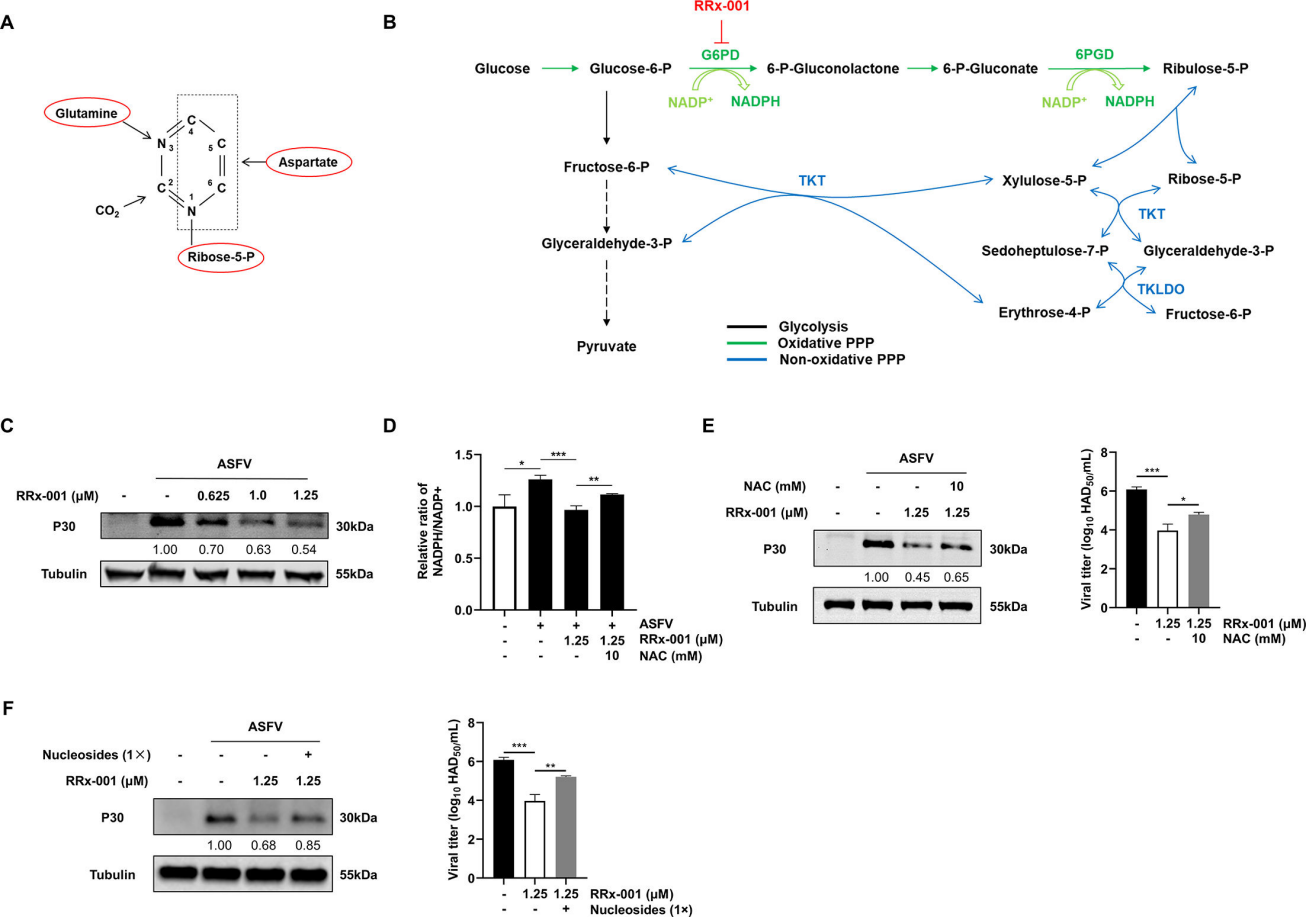
The PPP coordinates antiviral redox balance and nucleotide metabolism to promote ASFV replication. (**A**) Schematic diagram illustrating the structure of pyrimidine nucleotide and the origin of each atom in the pyrimidine ring. (**B**) Schematic diagram of glycolysis, the PPP, and the RRx-001 target site. RRx-001 is an inhibitor of G6PD, the rate-limiting enzyme of the PPP. (**C**) Effects of RRx-001 on ASFV-P30 protein expression levels. (**D**) Determination of intracellular NADPH/NADP^+^ ratio under different treatment conditions. (**E, F**) Western blot and virus titration analysis of ASFV replication in infected cells following the addition of exogenous NAC (an antioxidant) and nucleosides. For all experiments, PAMs were infected with ASFV at MOI = 1, and cell lysates were collected at 24 hpi for Western blotting. Statistical significance is indicated as follows: *P < 0.05, **P < 0.01, ***P < 0.001, n.s. = no significant difference (*P* ≥ 0.05).

Our results demonstrate that ASFV replication depends on *de novo* pyrimidine nucleotide synthesis, which involves two essential components: the provision of R5P and the construction of the pyrimidine ring (Fig. 3A). The PPP supplies R5P, a key precursor for nucleotide synthesis. To investigate the role of the PPP in ASFV replication, we treated infected cells with RRx-001 (Fig. 3B), an inhibitor of glucose-6-phosphate dehydrogenase (G6PD), the rate-limiting enzyme of the PPP. Dose-response assays revealed a significant reduction in ASFV-P30 protein expression (Fig. 3C), indicating that PPP activity is essential for ASFV replication. In addition, the oxPPP generates NADPH, which enhances viral antioxidant capacity and supports DNA synthesis (10). Our previous study found that ASFV infection increases intracellular ROS levels (11); therefore, we hypothesized that ASFV selectively hijacks the oxPPP to generate NADPH and establish a favorable environment for DNA synthesis under oxidative stress. Consistent with this hypothesis, infected cells exhibited elevated NADPH/NADP^+^ ratios (Fig. 3D), while RRx-001 treatment abrogated this increase (Fig. 3D), suggesting that viral replication requires oxPPP-derived NADPH to maintain redox homeostasis. To determine whether PPP supports ASFV replication via NADPH-mediated antioxidant defense, nucleotide precursor provision, or both, we performed functional rescue experiments with N-acetylcysteine (NAC, an antioxidant) and exogenous nucleosides. Both treatments partially restored viral replication following RRx-001 inhibition (Fig. 3E and F), indicating that the PPP facilitates ASFV replication through dual mechanisms: NADPH-dependent redox regulation and nucleotide precursor supply.

### ASFV replication depends on glutamine uptake and induces dynamic remodeling of the host glutamine pool

*De novo* pyrimidine nucleotide biosynthesis requires not only R5P, supplied by the PPP, but also glutamine, which donates the γ-nitrogen for pyrimidine ring assembly (Fig. 3A). This positions glutamine as a key metabolic checkpoint in ASFV replication.

**Fig 4:**
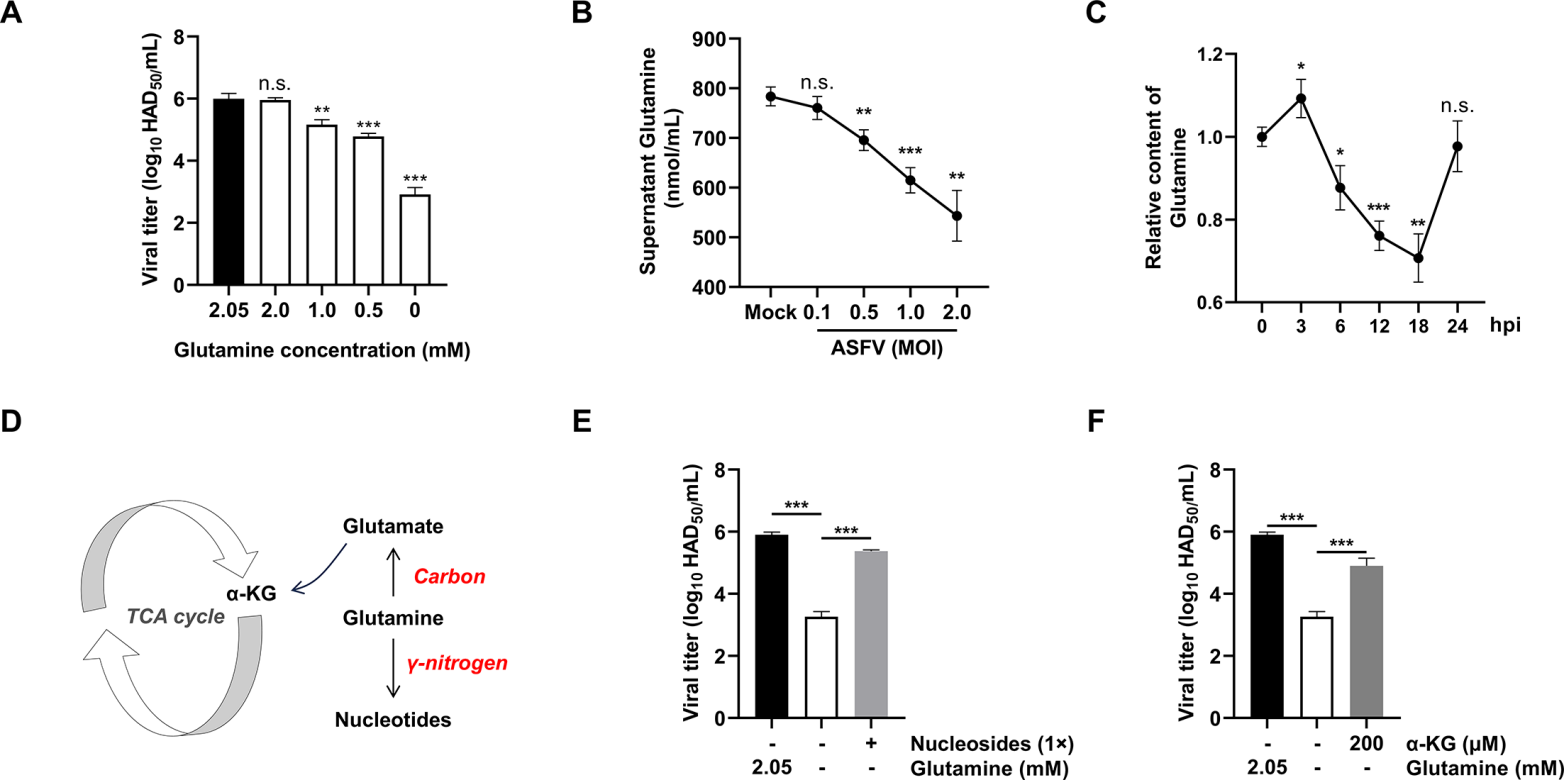
Exogenous glutamine is necessary for ASFV replication. (**A**) Impact of exogenous glutamine depletion on ASFV titer (normal culture medium containing 2.05 mM glutamine). (**B**) Determination of glutamine levels in the culture supernatant of PAMs infected with ASFV at different MOI for 24 h. (**C**) Determination of intracellular glutamine levels in PAMs infected with ASFV at 1 MOI at various time points. (**D**) Pathway diagram of glutamine catabolism. (**E, F**) Rescue of ASFV replication inhibition caused by exogenous glutamine deprivation through supplementation of exogenous nucleosides and α-ketoglutarate. Unless otherwise indicated, PAMs were infected with ASFV at MOI = 1, and samples were collected at 24 hpi for Western blotting, real-time quantitative PCR (RT-qPCR), and viral titer analysis. Statistical significance is indicated as follows: *P < 0.05, **P < 0.01, ***P < 0.001, n.s. = no significant difference (*P* ≥ 0.05).

To investigate the role of glutamine in ASFV replication, we cultured infected cells in glutamine-depleted medium, then supplemented them with varying concentrations of glutamine and assessed ASFV replication by measuring viral titers. The results showed a strong dependence on glutamine: ASFV titers were significantly reduced under glutamine-free conditions but restored upon glutamine supplementation (Fig. 4A). This confirms glutamine as an essential metabolic substrate for ASFV replication.

To further elucidate the impact of ASFV infection on glutamine utilization, we measured glutamine levels in both the culture supernatant and within cells following ASFV infection of PAMs. Our data showed that ASFV infection dose-dependently reduced glutamine concentrations in the culture supernatant (Fig. 4B). However, intracellular glutamine levels initially increased 3 hpi, followed by a marked decline (Fig. 4C). Collectively, these findings suggest that ASFV infection not only enhances the uptake of extracellular glutamine but also promotes glutamine catabolism during its replication.

### ASFV exploits dual glutamine metabolic pathways to sustain nucleotide biosynthesis and TCA cycle replenishment for viral replication

Glutamine serves as a critical source of both carbon and nitrogen for biosynthesis. Upon cellular uptake, its γ-nitrogen is directly utilized for nucleotide biosynthesis, while its carbon skeleton is converted into glutamate and further metabolized into α-KG to replenish the TCA cycle for energy and biosynthesis (Fig. 4D). To determine whether ASFV-induced glutamine catabolism operates through these pathways, we used glutamine-deficient medium as a baseline and supplemented it separately with either nucleosides or α-KG. Notably, nucleoside supplementation restored ASFV replication (Fig. 4E), demonstrating that glutamine-derived nitrogen supports *de novo* nucleotide synthesis essential for viral propagation. Concurrently, α-KG supplementation also restored viral replication efficiency (Fig. 4F), suggesting that glutamine-derived carbon flux may sustain TCA cycle activity, which could contribute to meeting biosynthetic and energetic demands. These findings collectively suggest that ASFV may redirect glutamine catabolism through two distinct pathways: one that directly incorporates nitrogen into nucleotide precursors and another that channels carbon into the TCA cycle via α-KG, thereby facilitating viral replication and maintaining host metabolic support.

### ASFV hijacks SLC1A5 to enhance glutamine-fueled nucleotide synthesis and drive viral proliferation

**Fig 5:**
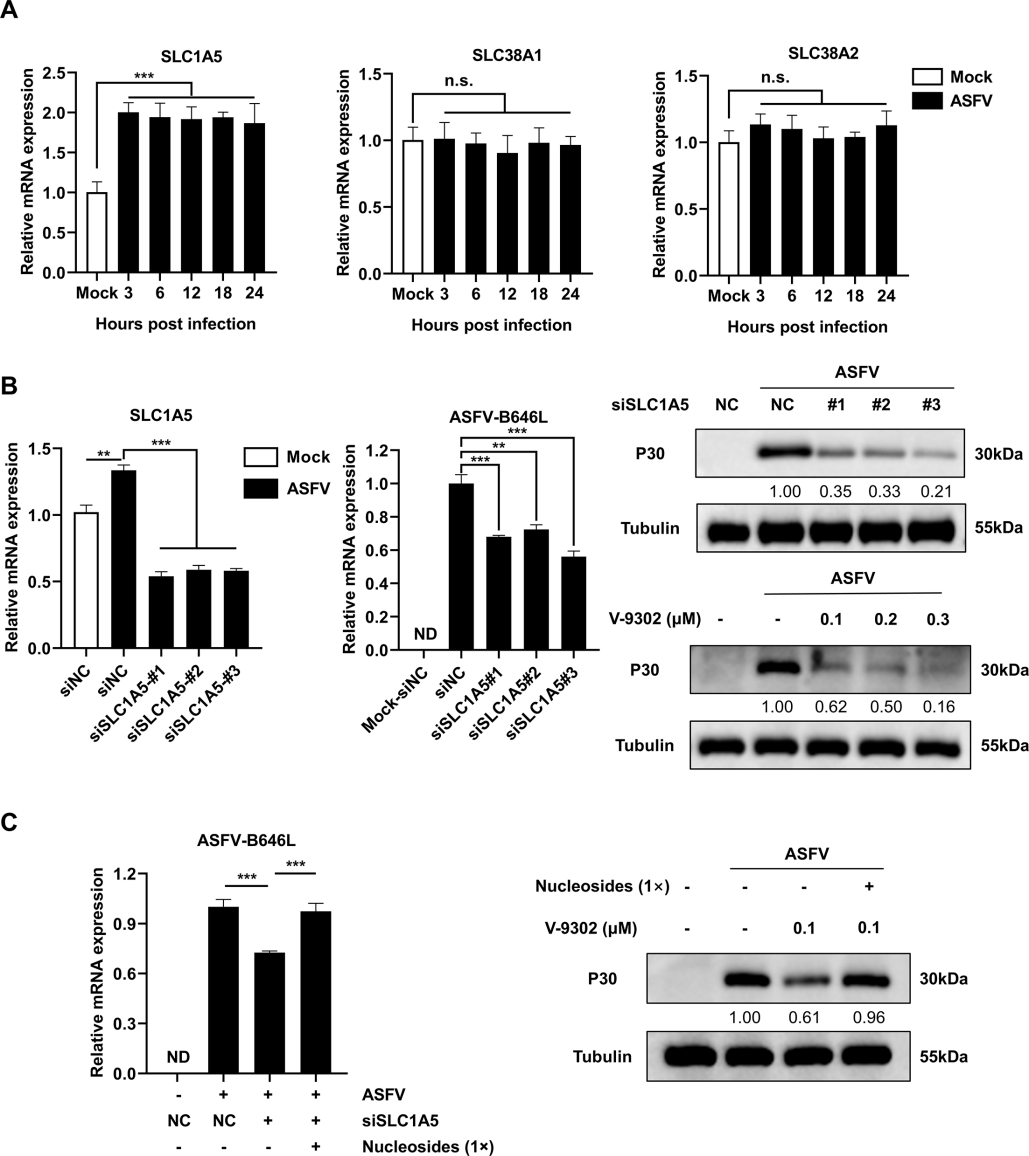
ASFV hijacks SLC transporter SLC1A5 to facilitate viral replication. (**A**) RT-qPCR analysis of mRNA levels of glutamine-related transporters in PAMs during ASFV infection at various time points. (**B**) Detection of the effects of SLC1A5 siRNA and inhibitor V-9302 on ASFV replication using RT-qPCR and Western blotting. (**C**) RT-qPCR and Western blot analysis of ASFV-B646L mRNA and ASFV-P30 protein expression in infected cells after the addition of exogenous nucleosides. Unless otherwise indicated, PAMs were infected with ASFV at MOI = 1, and samples were collected at 24 hpi for Western blotting, RT-qPCR, and viral titer analysis. Statistical significance is indicated as follows: *P < 0.05, **P < 0.01, ***P < 0.001, n.s. = no significant difference (*P* ≥ 0.05).

While our previous findings established ASFV’s reliance on glutamine catabolism, the mechanism underlying its enhanced glutamine uptake remained unclear. Given that glutamine transport is mediated by SLC proteins—membrane-bound transporters critical for amino acid homeostasis (21)—we hypothesized that ASFV might selectively upregulate glutamine-specific SLC transporters to meet its biosynthetic demands, similar to strategies employed by other viruses (22, 23). The primary glutamine transporters include SLC1A5, SLC38A1, and SLC38A2 (24). To determine which of these are involved in ASFV-induced glutamine metabolism, we evaluated the mRNA expression levels of these transporters during ASFV infection. Our results showed that SLC1A5 mRNA expression was significantly upregulated, whereas SLC38A1 and SLC38A2 expression remained unchanged (Fig. 5A). Moreover, treatment with the SLC1A5-specific inhibitor V-9302 or siRNA-mediated knockdown of SLC1A5 significantly inhibited ASFV replication (Fig. 5B; Fig. S3A and B). Importantly, supplementation with exogenous nucleoside restored viral replication under SLC1A5 inhibition (Fig. 5C), directly linking SLC1A5-driven glutamine uptake to nucleotide biosynthesis. Taken together, these findings suggest that ASFV-induced upregulation of SLC1A5 facilitates increased glutamine uptake, thereby supporting the nucleotide biosynthesis essential for viral replication.

### ASFV replication is unaffected by exogenous aspartate deprivation

**Fig 6:**
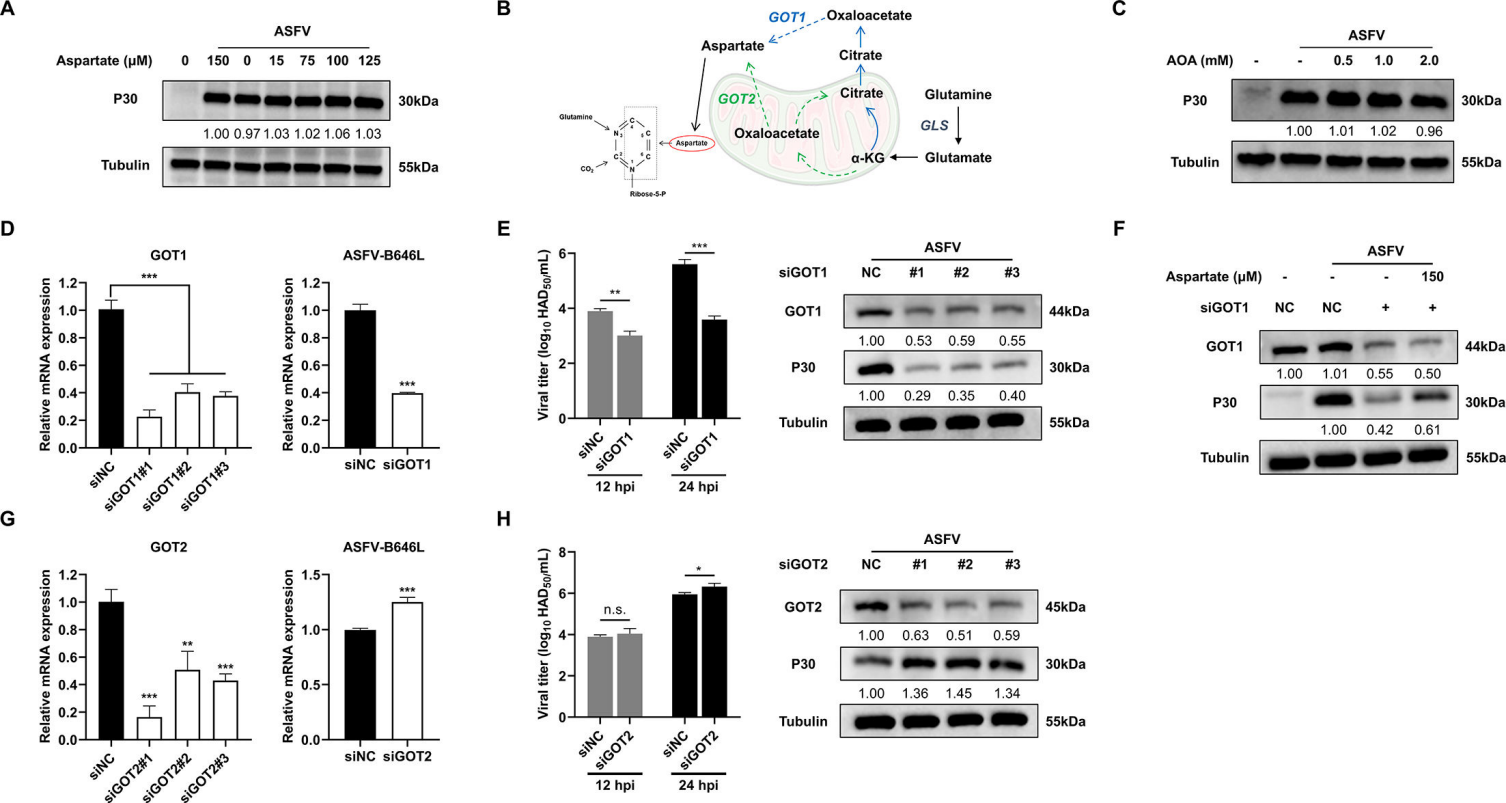
Endogenous aspartate synthesis regulates ASFV replication. (**A**) Exogenous aspartate deficiency does not affect the replication of ASFV (normal culture medium containing 150 µM aspartate). (**B**) Schematic diagram of endogenous synthesis pathway of aspartate. (**C**) Detection of the effect of aminooxyacetic acid hemihydrochloride (AOA) on the expression of ASFV-P30 protein using Western blotting. (**D**) RT-qPCR analysis of ASFV-B646L mRNA expression in infected cells treated with GOT1 siRNA. (**E**) Western blot and virus titration assessing the effect of GOT1 siRNA on ASFV-P30 expression and infectious virus production. (**F**) Exogenous aspartate supplementation restores ASFV-P30 protein levels suppressed by GOT1 siRNA. (**G**) RT-qPCR analysis of ASFV-B646L mRNA expression in infected cells treated with GOT2 siRNA. (**H**) Detection of the effect of GOT2 siRNA on the expression of ASFV-P30 protein expression and virus titer. For all experiments, PAMs were infected with ASFV at MOI = 1, and cell lysates were collected at 24 hpi for Western blotting. Statistical significance is indicated as follows: *P < 0.05, **P < 0.01, ***P < 0.001, n.s. = no significant difference (*P* ≥ 0.05).

Virus-induced alterations in amino acid metabolism provide insight into how viruses reprogram host resources to support replication. Previous studies have reported that ASFV disrupts normal amino acid metabolism, including that of aspartate (4). Aspartate is also a crucial substrate for pyrimidine ring assembly (Fig. 3A), and it can be supplied either through extracellular uptake or intracellular *de novo* synthesis. To preliminarily examine ASFV’s aspartate utilization strategy, we cultured infected cells in aspartate-deficient medium and performed supplementation experiments using varying concentrations of aspartate. Notably, the absence of exogenous aspartate did not affect the replication of ASFV (Fig. 6A; Fig. S3C through E), indicating that ASFV replication is resilient to extracellular aspartate deprivation. These findings suggest that ASFV may rely primarily on intracellular *de novo* aspartate synthesis to meet its replication needs.

### *De novo* aspartate synthesis sustains ASFV replication through compartmentalized metabolic regulation

Given ASFV’s independence from extracellular aspartate, we investigated whether viral replication relies on endogenous *de novo* aspartate biosynthesis. Two major pathways contribute to intracellular aspartate production. Specifically, glutamine is converted by glutaminase into glutamate and subsequently into α-KG. Within mitochondria, α-KG is processed through the TCA cycle to generate OAA, which is then converted to aspartate by mitochondrial GOT2. Alternatively, α-KG can undergo reductive carboxylation to form citrate, which is exported to the cytosol, converted into OAA, and finally transformed into aspartate by cytosolic GOT1 (Fig. 6B).

To examine the contribution of these pathways, we initially treated infected cells with aminooxyacetic acid hemihydrochloride (AOA), a pan-transaminase inhibitor. Unexpectedly, AOA at non-toxic concentrations did not significantly inhibit viral replication (Fig. 6C), suggesting that broad-spectrum transaminase inhibition alone is insufficient to disrupt the compensatory mechanisms regulating aspartate biosynthesis in infected cells. This suggests the presence of redundant transaminase activity or activation of alternative pathways, ensuring the continued aspartate production to support viral replication. To further dissect compartment-specific roles, we employed siRNA-mediated knockdown of GOT1 or GOT2. Knockdown of GOT1 markedly suppressed ASFV replication (Fig. 6D and E), and aspartate supplementation partially restored replication efficiency (Fig. 6F), confirming that cytosolic aspartate synthesis is critical for viral propagation. Paradoxically, silencing GOT2 enhanced viral replication (Fig. 6G and H), suggesting that inhibition of mitochondrial aspartate flux may promote viral proliferation through currently undefined mechanisms. Collectively, these findings demonstrate that ASFV replication critically depends on *de novo* aspartate synthesis, primarily via the cytosolic GOT1 pathway. However, the counterintuitive enhancement of replication following GOT2 suppression highlights a regulatory role for mitochondrial aspartate metabolism, underscoring the need for further investigation into the compartmentalized regulation of GOT1 and GOT2 during ASFV infection.

### Temporal reciprocal regulation of GOT1 and GOT2 directs aspartate flux essential for ASFV replication

**Fig 7:**
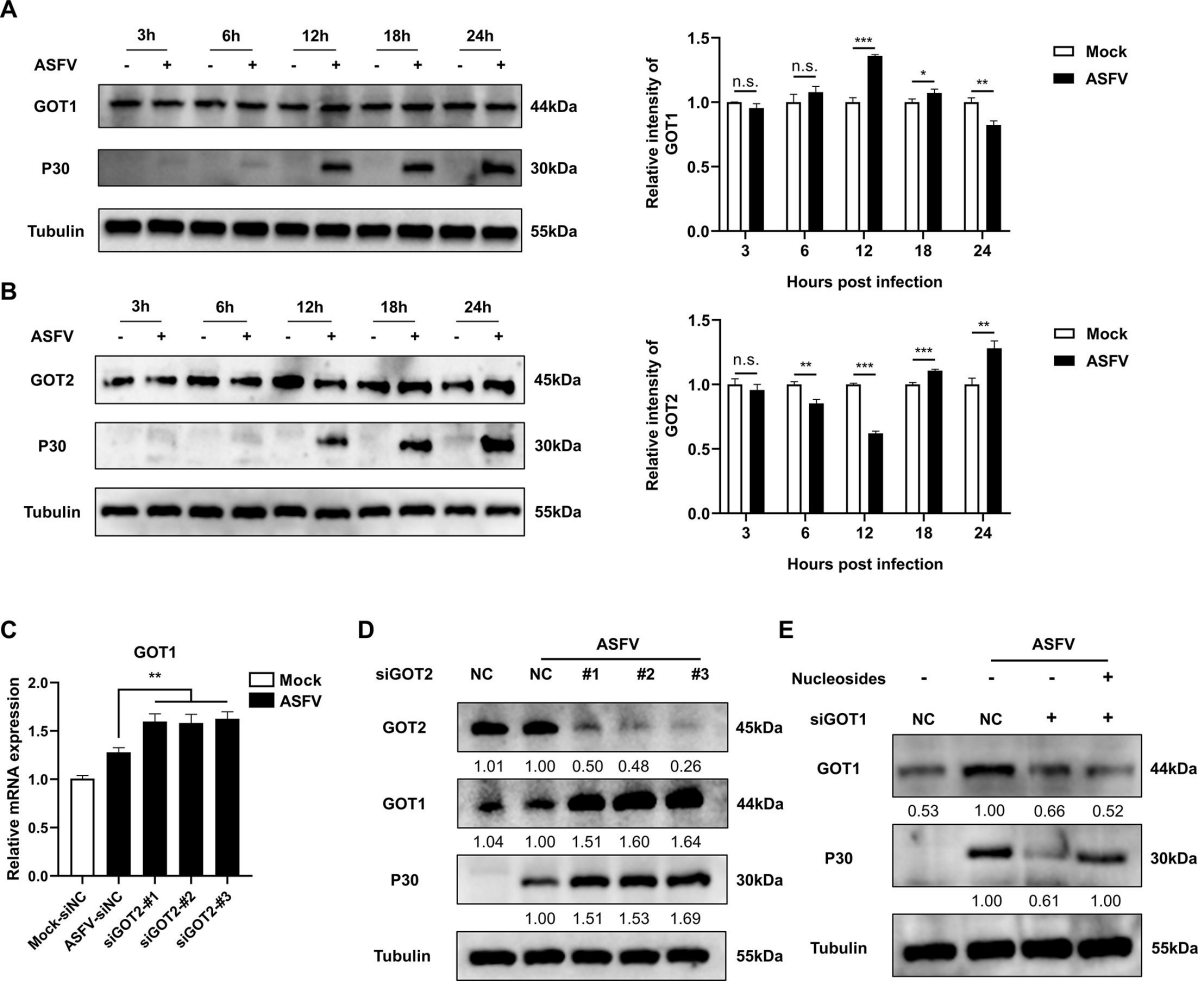
Temporal GOT1–GOT2 reciprocity directs aspartate flux for ASFV replication. (**A, B**) Expression levels of GOT1 and GOT2 during ASFV infection at various time points. (**C, D**) RT-qPCR and Western blot analysis of changes in GOT1 expression after GOT2 knockdown by siRNA. (**E**) Exogenous nucleoside supplementation restores ASFV-P30 protein levels suppressed by GOT1 siRNA. Unless otherwise indicated, PAMs were infected with ASFV at MOI = 1, and samples were collected at 24 hpi for Western blotting, RT-qPCR, and viral titer analysis. Statistical significance is indicated as follows: *P < 0.05, **P < 0.01, ***P < 0.001, n.s. = no significant difference (*P* ≥ 0.05).

To elucidate ASFV’s regulatory strategy over GOT1 and GOT2, we mapped the kinetics of their protein expression at various time points post-infection. As shown in Fig. 7A, GOT1 exhibited a biphasic expression pattern: levels gradually increased during early-to-mid infection (3–12 hpi), peaked at 12 hpi, and declined to basal levels during late infection (18–24 hpi). In contrast, GOT2 displayed an inverse trend, with its expression decreasing following infection and subsequently increasing after 12 hpi (Fig. 7B). This reciprocal expression pattern suggests that ASFV strategically prioritizes cytosolic aspartate synthesis via GOT1 during active genome replication (318 hpi) while simultaneously suppressing mitochondrial aspartate flux through GOT2 to minimize metabolic competition. Given that GOT1 knockdown inhibited ASFV replication, whereas GOT2 knockdown enhanced it, we further investigated potential functional crosstalk between these enzymes. Our results showed that siRNA-mediated silencing of GOT2 led to a compensatory upregulation of GOT1 (Fig. 7C and D), indicating that when mitochondrial aspartate production is impaired, ASFV may rebalance aspartate synthesis through the cytosolic route. This compensatory mechanism likely explains why GOT2 depletion paradoxically enhances ASFV replication, as enhanced GOT1 activity amplifies cytosolic aspartate production to support viral nucleotide biosynthesis. Finally, to directly link GOT1-derived aspartate to viral nucleotide biosynthesis, we performed an exogenous nucleoside supplementation assay following GOT1 knockdown using siRNA. As shown in Fig. 7E, exogenous nucleoside supplementation partially restored ASFV replication. This result confirms that GOT1-mediated aspartate synthesis primarily supports *de novo* nucleotide production rather than general metabolic homeostasis, underscoring its importance in ASFV replication.

## DISCUSSION

In this study, we elucidated how ASFV hijacks host nucleotide metabolism to support its replication by reprogramming key metabolic pathways. Our untargeted metabolomics analysis of ASFV-infected PAMs revealed significant perturbations in purine and pyrimidine metabolism, glycolysis, the PPP, and amino acid pathways, including those of glutamine and aspartate. Functional experiments confirmed that ASFV replication critically depends on *de novo* pyrimidine biosynthesis. Notably, our findings show that ASFV employs a dual strategy to secure nucleotide precursors: (i) it activates the PPP to generate R5P and maintain NADPH-mediated redox homeostasis, and (ii) it promotes glutamine uptake and catabolism to supply both the nitrogen and carbon required for nucleotide synthesis and TCA cycle replenishment. Furthermore, our results indicate that ASFV induces metabolic adaptations in aspartate synthesis, favoring intracellular production via cytosolic GOT1 while remaining resilient to extracellular aspartate deprivation. Collectively, these findings provide novel insights into the metabolic interplay between ASFV and its host cells and identify potential metabolic vulnerabilities that could be targeted for antiviral therapy.

Our results are consistent with numerous studies demonstrating that viruses reprogram host metabolic pathways to meet the demands of replication. Research on viruses such as Epstein-Barr virus, Kaposi’s sarcoma-associated herpesvirus, coxsackievirus B3, vaccinia virus, and has shown that these pathogens hijack host nucleotide biosynthesis for their replication (25–29). Similarly, ASFV appears to selectively manipulate host pyrimidine nucleotide metabolism. The observed dependence of ASFV on *de novo* pyrimidine synthesis is supported by our data showing that inhibition of DHODH with brequinar, but not inhibition of purine synthesis with AVN-944, significantly reduces viral replication. As for why ASFV replication tends to utilize the synthesis of pyrimidine nucleotides, we speculate that *de novo* purine nucleotide biosynthesis is more energy-intensive due to the complex assembly of cyclic structures and multi-step regulatory processes, whereas pyrimidine biosynthesis is relatively more energy-efficient. This distinction could play a pivotal role in the metabolic “tug-of-war” between ASFV and its host. Notably, our previous study demonstrated that brequinar inhibits ASFV replication by activating ferroptosis, a regulated form of cell death, highlighting its potential as an antiviral agent (30). However, that study did not address the metabolic prerequisites that render ASFV susceptible to DHODH inhibition. In contrast, the current work systematically dissects the virus-induced reprogramming of host pyrimidine metabolism, revealing how ASFV sustains *de novo* nucleotide biosynthesis through host pathways, thereby creating a state of metabolic dependency exploitable by brequinar. Together, these two findings support a “metabolic-cell death” collaborative model for brequinar’s antiviral mechanism against ASFV, providing a theoretical foundation for dual-targeted therapeutic strategies.

Moreover, the role of the PPP in maintaining redox balance and supplying nucleotide precursors has been well documented in the literature (31). Our study extends these observations to ASFV by demonstrating that inhibition of the PPP rate-limiting enzyme G6PD via RRx-001 not only diminishes nucleotide precursor production but also reduces the NADPH/NADP^+^ ratio, which is crucial for mitigating oxidative stress. Previous studies have indicated that an elevated NADPH/NADP^+^ ratio reflects a reductive intracellular environment that maintains redox homeostasis and facilitates DNA synthesis (28). Our results show that exogenous nucleoside or antioxidant supplementation partially rescues viral replication under G6PD inhibition, underscoring the dual role of the PPP in supplying nucleotide precursors and maintaining redox balance.

While our data support the contribution of oxPPP-derived NADPH to oxidative stress defense and suggest a role for the PPP in nucleotide synthesis, they do not conclusively demonstrate that ASFV specifically enhances nucleotide precursor flux through the oxPPP. Notably, [1,2-^13^C_2_]-glucose was used in this study to trace the metabolic flow of ^13^C in mock-infected and ASFV-infected cells. However, there was no significant difference in (M + 1)-labeled R5P flux between mock-infected and ASFV-infected cells (data not shown). This discrepancy may arise from insufficient detection sensitivity or compensatory R5P production via the non-oxPPP branch or salvage pathways, which merits further investigation.

Glutamine is a crucial amino acid, serving as both an important energy substrate and a key source of carbon and nitrogen for biosynthetic reactions. Our results are consistent with recent reports that viral infections can upregulate specific amino acid transporters to meet their biosynthetic and energetic demands (32). We observed that ASFV infection upregulates the glutamine transporter SLC1A5, which enhances glutamine uptake. This increased uptake directs glutamine’s nitrogen toward pyrimidine synthesis and its carbon toward TCA cycle replenishment to sustain metabolic flux. Notably, the rebound in intracellular glutamine levels at 24 hpi (Fig. 4C) may reflect a compensatory metabolic response. Initially, ASFV infection accelerates the uptake and catabolism of extracellular glutamine to satisfy high metabolic demands for nucleotide biosynthesis and TCA cycle replenishment, thereby depleting intracellular glutamine. However, the sudden increase observed at 24 hpi may indicate an upregulation of alternative glutamine uptake pathways or a reduction in glutamine consumption during the late stages of infection. This shift might serve to restore intracellular metabolic balance and ensure the availability of glutamine for critical biosynthetic processes, ultimately supporting sustained viral replication during advanced stages of infection. In addition, Dai et al. reported that during the late phase of ASFV infection, host cells produce abundant phenyllactic acid, which accumulates and inhibits the utilization of glutamine by ASFV, indirectly explaining the increase in intracellular glutamine levels observed at 24 hpi (33). Overall, our findings are consistent with previous reports linking elevated glutamine uptake to enhanced viral replication (34, 35).

Aspartate is recognized as a critical metabolite for cell proliferation due to its essential role in nucleotide synthesis (36). Although previous studies have highlighted the importance of extracellular aspartate in supporting viral replication (19), our data reveal that ASFV bypasses this dependency by activating GOT1-mediated, cell-autonomous aspartate synthesis. Additionally, we observed that suppression of mitochondrial GOT2 paradoxically enhances viral replication. Previous reports have demonstrated that cytosolic GOT1 and mitochondrial GOT2 are co-regulated by the mitochondrial electron transport chain (ETC). Specifically, impairment of ETC complex I (NADH dehydrogenase) disrupts mitochondrial aspartate synthesis, thereby triggering a compensatory increase in cytosolic aspartate production via GOT1 (16, 17). Mitochondria, particularly complexes I and III of the ETC, are major sources of ROS, and excessive ROS can inflict oxidative damage on key ETC components, including complex I (37). Studies have shown that ASFV infection induces significant ROS production and oxidative stress (38, 39). Our findings align with this model: GOT2 knockdown results in a compensatory increase in GOT1 expression, rerouting aspartate synthesis to the cytosol. ASFV, which replicates in cytoplasmic viral factories, likely benefits from localized aspartate pools that directly feed viral nucleotide biosynthesis.

**Fig 8:**
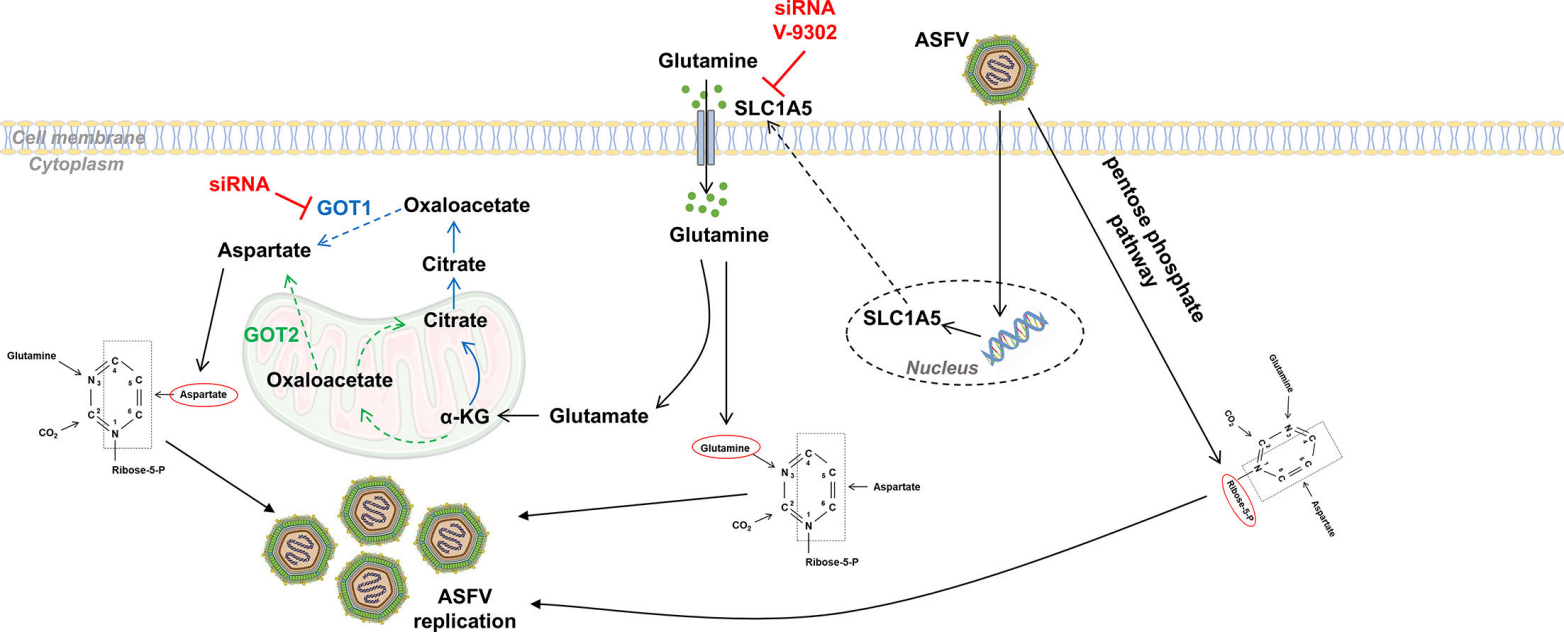
Schematic diagram illustrating the mechanisms by which ASFV regulates nucleotide synthesis precursors through multiple pathways. ASFV reprograms host central carbon and nitrogen metabolism to fuel *de novo* pyrimidine nucleotide synthesis. First, ASFV activates the PPP, diverting glucose-derived flux to generate R5P, which is essential for nucleotide backbone synthesis. Second, ASFV upregulates the expression of the glutamine transporter SLC1A5, enhancing cellular glutamine uptake. Imported glutamine serves dual roles: (i) as a nitrogen donor for nucleotide biosynthesis, and (ii) as a carbon source, being converted to α-KG via glutaminolysis. α-KG enters the TCA cycle and is further converted by GOT1 to produce aspartate, which is required for pyrimidine ring formation. Together, these pathways ensure an adequate supply of nucleotide precursors to support ASFV DNA replication and gene expression. Viral hijacking of host metabolism thus represents a coordinated strategy to sustain robust viral replication.

In conclusion, our findings support a model in which ASFV orchestrates coordinated reprogramming of host metabolism, specifically targeting nucleotide biosynthesis to create an environment favorable to viral replication. By simultaneously enhancing the PPP, upregulating glutamine uptake and metabolism, and rerouting aspartate synthesis to the cytosol, ASFV ensures a robust supply of both energy and essential biosynthetic precursors (Fig. 8). Furthermore, our work extends the understanding of ASFV metabolic hijacking by highlighting its selective reliance on pyrimidine synthesis, which may reflect evolutionary pressures favoring energy-efficient pathways under conditions of high metabolic competition. This dual strategy—targeting both the supply of nucleotide precursors and the maintenance of redox balance—underscores the sophistication of ASFV’s metabolic adaptations and provides a compelling rationale for therapeutic targeting of these pathways.

## MATERIALS AND METHODS

### Virus, cell lines, antibodies, and chemical reagents

The ASFV strain GZ201801_2 (GenBank accession number ON263123) was isolated from clinical specimens during the early ASF outbreaks in 2018 and is preserved at the Infectious Diseases Laboratory of South China Agricultural University. PAMs were isolated from 28-day-old specific-pathogen-free pigs and maintained at 37°C with 5% CO_2_ in RPMI 1640 medium (Gibco, Billings, MT, USA) supplemented with 10% fetal bovine serum (Gibco).

The antibodies used in this study included ASFV-P30 mouse monoclonal antibody (produced by our laboratory), β-tubulin (M20005; Abmart), GOT1 (14886-1-AP; Proteintech), and GOT2 (67738-1-Ig; Proteintech). The chemical reagents used were AVN-944 (HY-13560), brequinar (HY-108325), NAC (HY-B0215), V-9302 (HY-112683), AZD-8055 (HY-10422), uridine (HY-B1449), and AOA (HY-107994), all from MedChemExpress. Glutamine (C0212) and aspartate (ST1476) were obtained from Beyotime. RRx-001 (S8405) was purchased from Selleck, and nucleosides (ES-008-D) from Sigma-Aldrich.

### siRNA transfection

siRNA transfection was performed using Lipofectamine RNAiMAX (Thermo Fisher Scientific) following the manufacturer’s instructions. The siRNA used in this study was designed and synthesized by Tsingke Biotech Co., Ltd. (Beijing, China). Briefly, 5 µL siRNA and 3 µL Lipofectamine RNAiMAX were each diluted in 100 µL Opti-MEM (31985070, Gibco). After a 5 min incubation at room temperature (RT), the two mixtures were combined, thoroughly mixed, and incubated for an additional 20 min at RT. The resulting transfection complex (200 µL/well) was added to 12-well culture plates and incubated for 24 h before proceeding with subsequent experimental procedures. The siRNA sequences are provided in Table 1.

**TABLE 1:** siRNA sequences targeting GOT1, GOT2, and SLC1A5

Name	Sense strand (5′–3′)	Antisense strand (5′–3′)
si-GOT1-1	GCACGAUGGUACAAUGGAA(dT)(dT)	UUCCAUUGUACCAUCGUGC(dT)(dT)
si-GOT1-2	CCAUGAAGCUGUCACCAAA(dT)(dT)	UUUGGUGACAGCUUCAUGG(dT)(dT)
si-GOT1-3	GCUAAUGACAGCAGCCUAA(dT)(dT)	UUAGGCUGCUGUCAUUAGC(dT)(dT)
si-GOT2-1	GGGUGGAGUCACAGUUGAA(dT)(dT)	UUCAACUGUGACUCCACCC(dT)(dT)
si-GOT2-2	GAGAGACACCAACAGCAAA(dT)(dT)	UUUGCUGUUGGUGUCUCUC(dT)(dT)
si-GOT2-3	GAGAUGUCUUCCUGCCGAA(dT)(dT)	UUCGGCAGGAAGACAUCUC(dT)(dT)
si-SLC1A5-1	GAAGGAAUCAGUCAUGUAA(dT)(dT)	UUACAUGACUGAUUCCUUC(dT)(dT)
si-SLC1A5-2	GCAAGAUUGUGGAGAUGGA(dT)(dT)	UCCAUCUCCACAAUCUUGC(dT)(dT)
si-SLC1A5-3	CAGUCAACCUACCAGUUCA(dT)(dT)	UGAACUGGUAGGUUGACUG(dT)(dT)

### Virus infection and drug treatment

PAMs were seeded into cell culture plates for 4 h to allow adherence, then washed with RPMI 1640 and incubated with an ASFV suspension (MOI = 1) for 2 h. After incubation, the supernatant was discarded and replaced with drugs at the appropriate concentrations depending on the experimental condition. At the indicated time points, cell samples were collected for real-time quantitative PCR (RT-qPCR), Western blotting, or other analyses. Unless otherwise indicated, PAMs were infected with ASFV at MOI = 1, and samples were collected at 24 hpi for Western blotting, RT-qPCR, and viral titer analysis.

### Hemadsorption assay

The hemadsorption assay was used to determine virus titers. The virus of unknown titer was serially diluted 10-fold in RPMI 1640 and inoculated onto PAMs seeded in 96-well plates. After 24 h, 15 µL of a 1% suspension of washed porcine erythrocytes was added to each well, and hemadsorption was recorded for up to 72 h. Endpoint titers were calculated using the Reed-Muench method.

### RT-qPCR

Total RNA was extracted from cell samples using the Total RNA Rapid Extraction Kit (220010; Fastagen) and reverse transcribed using the StarScript III RT Kit (A232-10; Genstar). qPCR was conducted using the ChamQ SYBR qPCR Master Mix (Q311-02; Vazyme) on a Bio-Rad CFX96 system. The primer sequences are provided in Table 2.

**TABLE 2:** Primers used in this study

Name	Sequence (5′–3′)
ASFV-B646L-F	ATAGAGATACAGCTCTTCCG
ASFV-B646L-R	GTATGTAAGAGCTGCAGAC
GOT1-F	GACAATGGCTGACCGCATTC
GOT1-R	TTCAACCTGCTTGGGGTTCA
GOT2-F	ATGGGCTTATACGGTGAGCG
GOT2-R	CGTTGACAGGAGGGTTGGAA
SLC1A5-F	AAAGACGGCTGCTGCGGTTC
SLC1A5-R	ACTGCCACCACGGTCAGGAG
SLC38A1-F	GTTGATCTGTTCAAAGGAAACA
SLC38A1-R	GCTCAGCATTGCTCCA
SLC38A2-F	ACTGTCTATGCTGTGCCAATTCTG
SLC38A2-R	CTTGGACACATTCATCATTCTTCTACG
GAPDH-F	CCTTCCGTGTCCCTACTGCCAAC
GAPDH-R	GACGCCTGCTTCACCACCTTCT

### Western blotting

Cell samples were collected and lysed in ice-cold RIPA lysis buffer (P0013B; Beyotime) for 10 min. Lysates were centrifuged at 4°C for 15 min, and total protein concentration in the supernatant was determined using the BCA Protein Assay Kit (P0012; Beyotime). Protein samples were separated using 10% SDS-PAGE and transferred onto a nitrocellulose (NC) membrane (Merck KGaA, Darmstadt, Germany). The NC membrane was blocked with 5% skim milk for 45 min and washed with Tris-buffered saline with 0.1% Tween 20 (TBST), followed by incubation with the primary antibody at 4°C for 8 h. The membrane was then incubated with the secondary antibody at RT for 1 h. Proteins were detected using a Tanon-5200 multi-infrared imaging system (Shanghai Tianneng Technology Co., Ltd., Shanghai, China). Each Western blot was repeated on lysates from three independent cell cultures (biological replicates).

### Cell viability, glutamine, and NADPH determination

Cell viability was assessed using the Cell Counting Kit-8 (C0038; Beyotime) after drug treatment. Glutamine levels were measured with the Glutamine Content Detection Kit (ml092936; MLBio), and the NADPH/NADP^+^ levels were determined using the CheKine Micro Coenzyme II NADP(H) Assay Kit (KTB1010; Abbkine). All experimental procedures were performed according to the manufacturer’s instructions.

### Statistical analysis

Metabolic pathway enrichment analysis of the metabolomics data was conducted using the online software MetaboAnalyst version 5.0 (www.metaboanalyst.ca). All data were analyzed using GraphPad Prism version 8.0 (GraphPad Software), and results are expressed as mean ± standard deviation from at least three independent experiments. Statistical significance was determined using Student’s *t*-test. *P < 0.05, **P < 0.01, and ***P < 0.001 were considered statistically significant.

## Data Availability

All data supporting the findings of this study are included in the article and its supplemental material. The processed metabolomics data sets generated in this study are provided as supplemetal material. Raw data are available from the corresponding author upon reasonable request.

## SUPPLEMENTAL MATERIAL

Supplemental figures jvi.00985-25-s0001.pdf Fig. S1 to S4.

Metabolomics data jvi.00985-25-s0002.xlsx Processed metabolomics datasets of ASFV-infected and mock-infected porcine alveolar macrophages at different time points post-infection. Each Excel sheet contains annotated metabolite information.

ASM does not own the copyrights to Supplemental Material that may be linked to, or accessed through, an article. The authors have granted ASM a non-exclusive, world-wide license to publish the Supplemental Material files. Please contact the corresponding author directly for reuse.
