# Supplementary material for: African swine fever virus hijacks host pyrimidine metabolism to promote viral replication
Source: J Virol. 2025 Oct 31;99(11):e00985-25. doi: 10.1128/jvi.00985-25 (PMC12645960; doi:10.1128/jvi.00985-25)
Supplement: Supplemental figures — Fig. S1 to S4. [file jvi.00985-25-s0001.pdf]

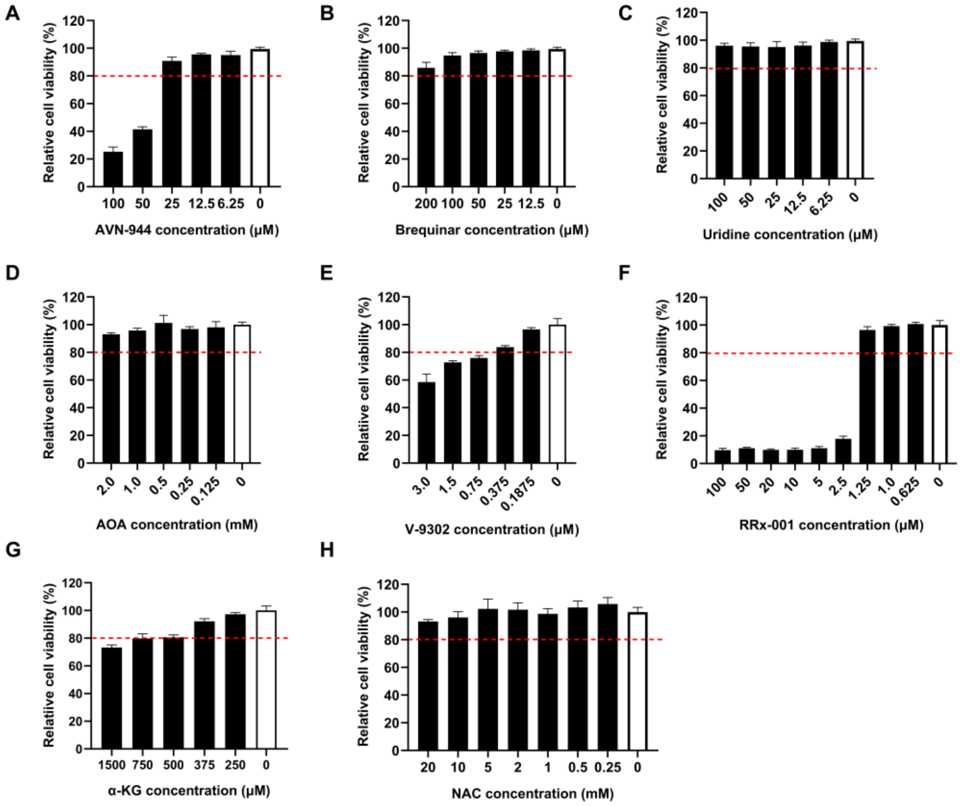

Fig S1 Assessment of cytotoxicity of different compounds in porcine alveolar macrophages (PAMs). PAMs were treated with various concentrations of (A) AVN-944, (B) Brequinar, (C) Uridine, (D) AOA, (E) V-9302, (F) RRx-001, (G) α-KG and (H) NAC for 24 hours. Cell viability was measured using the CCK-8 assay, and results are presented as the percentage of viable cells relative to untreated controls.

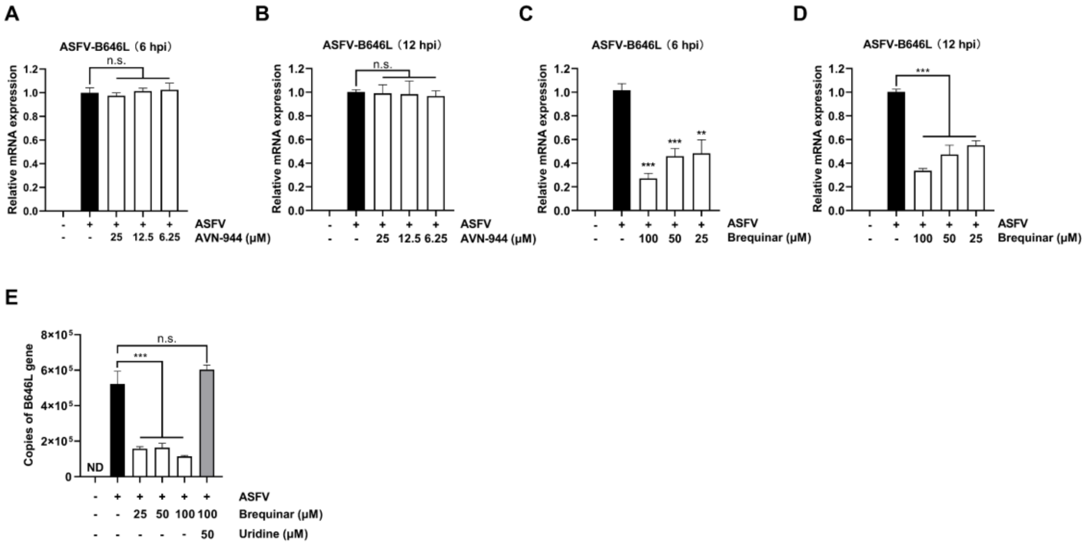

Fig S2 Effects of AVN-944 and brequinar on ASFV replication. PAMs were infected with ASFV at MOI = 1 and treated with various concentrations of AVN-944 (A, B) and brequinar (C, D), ASFV-B646L levels were measured by RT-qPCR (normalized to GAPDH). (E) The copies of the ASFV-B646L gene in infected PAMs was detected by quantitative PCR. Unless otherwise indicated, PAMs were infected with ASFV at MOI = 1, and samples were collected at 24 hpi for RT-qPCR and viral titer analysis.

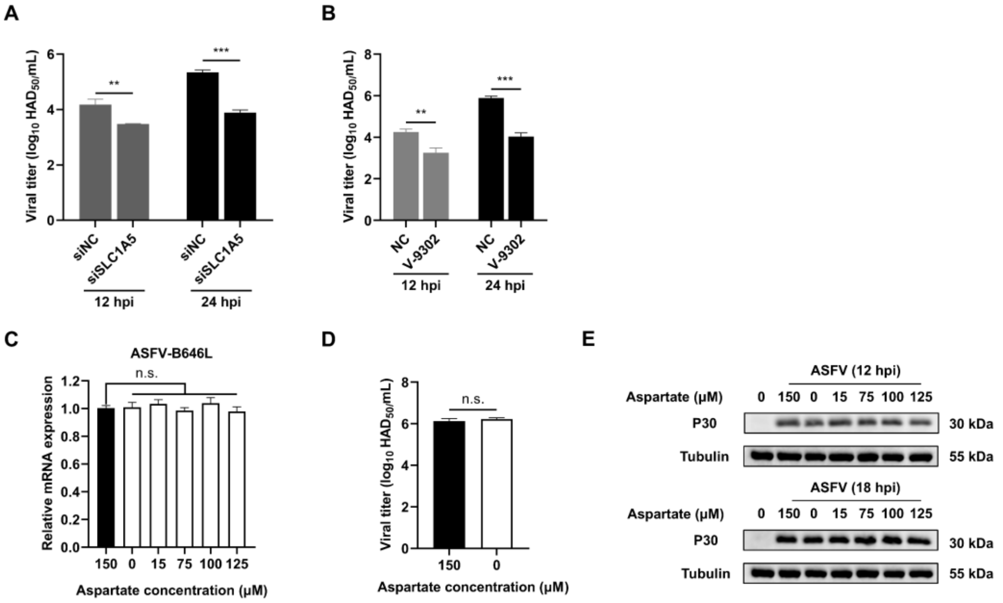

Fig S3 The effects of different treatment methods on the replication of ASFV. (A) Knockdown of SLC1A5 using siRNA significantly reduced virus titers compared to negative control (siNC), measured at 12 and 24 hpi. (B) Treatment with the SLC1A5 inhibitor V-9302 also led to decreased virus titers at both 12 and 24 hpi. (C) RT-qPCR, (D) viral titer and (E) Western blotting analysis of ASFV replication in PAMs cultured in aspartate-deprived medium. Unless otherwise indicated, PAMs were infected with ASFV at MOI = 1, and samples were collected at 24 hpi for Western blotting, RT-qPCR and viral titer analysis.

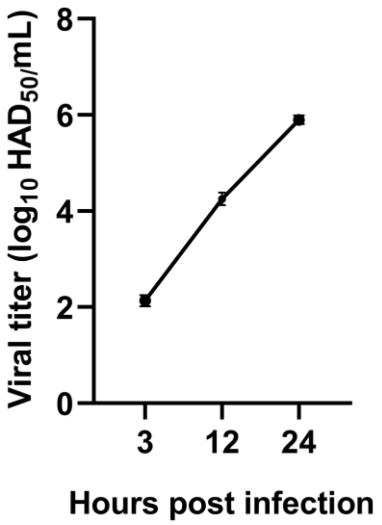

Fig S4 Growth curve replication of the GZ201801_2. PAMs were infected with ASFV (GZ201801_2) at MOI = 1, and the HAD50 (50% hemadsorption doses) of samples at different time point was measured.
